# “I Had Bills to Pay”: a Mixed-Methods Study on the Role of Income on Care Transitions in a Public-Payer Healthcare System

**DOI:** 10.1007/s11606-023-08024-7

**Published:** 2023-01-25

**Authors:** Muskaan Sachdeva, Amy Troup, Lianne Jeffs, John Matelski, Chaim M. Bell, Karen Okrainec

**Affiliations:** 1 Faculty of Medicine, University of Toronto, Toronto, Ontario Canada; 2 Toronto General Hospital Research Institute, University Health Network, Toronto, Ontario Canada; 3 Lunenfeld-Tanenbaum Research Institute Sinai Health, Toronto, Ontario Canada; 4 Biostatistics Research Unit, University Health Network, Toronto, Ontario Canada; 5 Department of Medicine, Sinai Health System, Toronto, Ontario Canada; 6 Institute of Health Policy, Management and Evaluation, University of Toronto, Toronto, Ontario Canada; 7 Department of Medicine, University Health Network, Toronto, Ontario Canada; 8 Toronto Western Hospital, 399 Bathurst Street, 8EW-408, Toronto, Ontario M5T 2S8 Canada

**Keywords:** disparities, socioeconomic, discharge, patient experience, readmission

## Abstract

**Background:**

Income disparities may affect patients’ care transition home. Evidence among patients who have access to publicly funded healthcare coverage remains limited.

**Objective:**

To evaluate the association between low income and post-discharge health outcomes and explore patient and caregiver perspectives on the role of income disparities.

**Design:**

Mixed-methods secondary analysis conducted among participants in a double-blind randomized controlled trial.

**Participants:**

Participants from a multicenter study in Ontario, Canada, were classified as low income if annual self-reported salary was below $29,000 CAD, or between $30,000 and $50,000 CAD and supported ≥ 3 individuals.

**Main Measures:**

The associations between low income and the following self-reported outcomes were evaluated using multivariable logistic regression: patient experience, adherence to medications, diet, activity and follow-up, and the aggregate of emergency department (ED) visits, readmission, or death up to 3 months post-discharge. A deductive direct content analysis of patient and caregivers on the role of income-related disparities during care transitions was conducted.

**Key Results:**

Individuals had similar odds of reporting high patient experience and adherence to instructions regardless of reported income. Compared to higher income individuals, low-income individuals also had similar odds of ED visits, readmissions, and death within 3 months post-discharge. Low-income individuals were *more* likely than high-income individuals to report understanding their medications completely (OR 1.9, 95% CI: 1.0–3.4) in fully adjusted regression models. Two themes emerged from 25 interviews which (1) highlight constraints of publicly funded services and costs incurred to patients or their caregivers along with (2) the various ways patients adapt through caregiver support, private services, or prioritizing finances over health.

**Conclusions:**

There were few quantitative differences in patient experience, adherence, ED visits, readmissions, and death post-discharge between individuals reporting low versus higher income. Several hidden costs for transportation, medications, and home care were reported however and warrant further research.

**Supplementary Information:**

The online version contains supplementary material available at 10.1007/s11606-023-08024-7.

## INTRODUCTION

An effective hospital-to-home transition is essential for optimizing post-discharge outcomes in patients.^1, 2^ Breakdowns in communication at hospital discharge can lead to fragmentation of care, poor understanding of discharge plans, adverse drug events, and higher readmission rates and costs.^3–5^ When compared to transitions to other facilities, care transition from hospital to home is associated with a 9–20% higher risk of early readmission or visit to the emergency department (ED).^6^ Care transitions from hospital to home thus merit the attention of healthcare systems, patients, and their caregivers, and are the focus of recent quality initiatives worldwide.^4, 7–9^

Though quality standards for care transitions exist for healthcare organizations, little attention has focused on the impact socioeconomic disparities may have on the transition from hospital to home. Current evidence on the association between SES and post-discharge outcomes is conflicting, with a few studies demonstrating higher length of stay, risk of ED visits, and of 30-day readmissions among individuals admitted with unique conditions such as pneumonia, heart failure, or cancer, while others demonstrate no association.^10–16^ This may be due to a paucity of research on patient-reported costs and how income disparities may affect health outcomes beyond readmissions.^12^

Current US-based research highlights challenges might relate to costs of filling prescriptions, affording sufficient home care, or adhering to follow-up appointments when they may lack transportation or have work commitments.^5, 17–20^ While publicly funded healthcare coverage exists in Canada to cover hospital care, primary and subspecialty outpatient physician care, and home and community care, recent research is pointing to insufficient public resources to meet the rising demand particularly related to home and community care.^21, 22^

The primary objective of our mixed-methods study was therefore to evaluate the association between low income and patient experience, adherence to instructions, and composite of ED visits, readmission, and death during the 3 months following discharge in a public-payer health setting of Canada. Our secondary objective was to explore patient and caregiver perspectives on costs in the post-discharge period and the association with health outcomes.

## METHODS

This article was written in accordance with the standards for reporting qualitative research (SRQR) including ensuring salient contextual factors, researcher characteristics, and member checking and triangulation was utilized.^23^

### Study Design

A mixed-methods secondary analysis was performed on eligible participants who were enrolled in a randomized control trial (RCT) evaluating the impact of a written discharge instruction plan on patient experience, adherence to instructions, and unscheduled visits and death.^24^ Individuals in the intervention group received an enhanced written plan with the diagnosis, instructions on diet or activity restrictions, what to do if problems arose, and who to see in follow-up. Our intervention did not include any discussion on costs and there were no differences in post-discharge outcomes between individuals in the intervention or arm who received usual discharge instructions (not yet published).^25^ In this context, the RCT provided a strong platform for conducting secondary data analysis on the impact of low income and costs on patient experience and other health outcomes following a discharge from hospital.

### Setting

Our study took place in Ontario, Canada, where permanent residents have publicly funded health insurance which covers hospital care, primary and subspecialty outpatient care, home and community care, and, for individuals 65 years and older, publicly funded medications. The participants enrolled in the RCT had been admitted to an acute care or rehabilitation hospital with congestive heart failure (CHF), chronic obstructive pulmonary disease (COPD), pneumonia, stroke, or an orthopedic procedure (hip fracture, hip replacement, or knee replacement), which are seven diagnoses associated with volume-based hospital funding. Participants who were not discharged home due to in-hospital death or transfer to other services or institutions were excluded. Participants who did not have outcome data due to being lost to follow-up were excluded from this study.

### Exposure Assessment

Participants were stratified in two groups based on their response to two questions regarding total annual household income and number of individuals supported by the income.^24, 26^ Individuals were assigned to the low-income group if their annual salary was below $29,000 CAD, or between $30,000 and $50,000 CAD if it supported three or more individuals (Appendix A).^27^ This measure has been supported to be a better indicator of poverty in Canada than studies, who until recently, used neighborhood quintile.^26^

### Quantitative Analytical Plan

We used the top box positive response to five patient experience measures from a standardized and validated survey collected via phone call between 72 h and 1 month following discharge (Appendix B). The Canadian Patient Experience Survey-Inpatient Care (CPES-IC) addresses receipt and understanding of discharge instructions as adapted from the Hospital Consumer Assessment of Healthcare Providers and Systems (HCAHPS) survey.^28^ A sixth question addressing understanding of the post-discharge follow-up plan was added given the importance of post-discharge follow-up on further ED visits, readmissions, and death.^17^ Patients were also asked about their adherence to discharge medications, diet or activity restrictions and follow-up appointments, and lastly, whether they had gone to the ED or been readmitted. Through chart review, we confirmed whether death had occurred at 1 and 3 months following discharge when family caregivers could not be reached.

### Statistical Methods

Demographic data collected included sex, age, admission diagnosis, self-reported disabilities, health literacy, language barrier, immigrant status, education level, and reliance on family for help with activities and instrumental activity of daily living such as self-care, food preparation, medications, transportation, and attending appointments.

The association between income group and post-discharge outcomes was evaluated using generalized linear mixed effects models, to allow for adjusted estimation of income group effects while accounting for site level clustering of responses via site level random intercepts. Variables included for adjustment were selected a priori based on clinical relevance and prior research. A binomial likelihood and logit link function were used, as implemented in R package lme4.^29^ Outcome variables with fewer than 30 events were not considered for modeling. For each outcome included, we report adjusted odds ratios (ORs) for the “low-income group” compared to the higher income group, along with 95% confidence intervals. Variables identified as potentially influencing the association between income and post-discharge outcomes (i.e., effect modifiers) were identified qualitatively and then assessed for statistical significance by augmenting the adjusted model with the candidate interaction terms (one at a time) and comparing the augmented model fit with the original model via a Likelihood Ratio Test. We include any significant interactions in the final models. A sensitivity analysis was conducted by including those who preferred not to respond to income category in our adjusted generalized linear mixed effects models. Lastly, we addressed the potential risk of selection bias from excluding individuals who were lost to follow-up by comparing demographic variables between excluded and included patients.

### Qualitative Analytical Plan

All participants enrolled in the RCT who were interested in the qualitative interview were contacted for consent for the study with enrolment stopping once thematic saturation had been met. Two sets of standardized semi-structured open-ended interview guides were used, one for patients and one for family caregivers, with a question focused on sociodemographic disparities. The influence of income, costs, and income-related disparities on post-discharge outcomes was addressed through patient and caregiver experiences during one-on-one telephone interviews conducted in English by one of our study authors (AT) and a separate research team supervised by the principal investigator (PI) (KO).

Recordings of semi-structured interviews were transcribed verbatim and these transcripts served as primary units of analysis using NVivo software. Given our qualitative study was focused more generally on patient experiences in the post-discharge period, we used a wide range of keywords to extract quotes to ensure a wider capture of relevant data for our research question. The following keywords were used by the lead author (MS) and reviewed by the PI (KO) to ensure they were reflective of our objectives: money, fortune, bills, food, transportation, emergency, groceries, activity, CCAC (community care access center), personal support worker (PSW), life, accommodation, afford, bank, budget, medication, refills, prescription, pension, disability, price, cost, resources, and salary. The research team consisted of a medical student conducting a graduate diploma in health research (MS), two internal medicine physicians and scientists with expertise in care transitions (KO and CB), a qualitative expert with an expertise in care transitions (LJ), and the study’s research coordinator (AT). Scripts of identified quotes from NVivo were reviewed independently by two reviewers (AT and MS) and any discordances reviewed by the principal investigator (KO) with input from our qualitative expert (LJ) when needed.

Directed content analysis was used where the major ideas emerging from reading the scripts were recorded and used to develop codes. With a direct content analysis, there were a priori concepts that helped organize our narrative though inductive analysis added methodological rigor to allow new codes to emerge. All coding was performed by two reviewers independently (MS, AT) and codes were discussed by all members of the qualitative team (MS, AT, KO, LJ). A deductive approach was then used to analyze retained scripts, independently, by all three reviewers (AT, MS, KO), for common themes. A fourth member (LJ) reviewed disagreements and discussions occurred until consensus was reached. A final step of reviewing codes by income category was completed (AT, MS, KO) in order to identify any differences in themes between low income, higher income, and preferred not to respond to income category.

### Ethics Approval

This study was approved by the institutional review board at the University Health Network, Thunder Bay Regional Health Sciences Centre, Baycrest Health Sciences Centre, Sinai Health System, and Bruyère Continuing Care.

## RESULTS

### Quantitative Results

**Figure 1:**
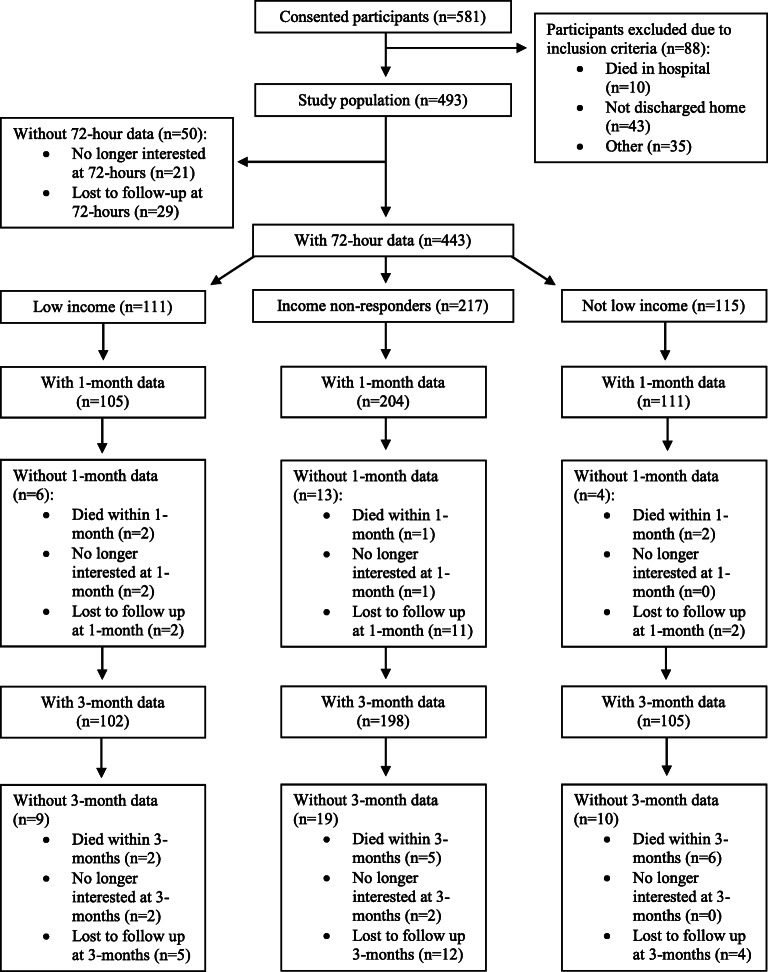
Methodology flowchart.

Out of the 581 participants that consented for the original RCT, 443 participants were included in this study. Eighty-eight were excluded due to becoming ineligible prior to discharge (i.e., were not discharged home due to death or transfer) and 50 were excluded as they were lost to follow-up. Based on self-reported income, 111 classified as low income, 115 reported higher income, and 217 did not know or preferred not to report their income status (Fig. 1). Individuals lost to follow-up were similar to those who remained in the analysis except that there were lower proportions who had been discharged following an orthopedic procedure (34% among study cohort vs. 10% among lost to follow-up, *p* < 0.001) (Appendix C).

Individuals who reported lower income were more likely to be older, less likely to have been admitted for an orthopedic procedure, and had a higher proportion with a language barrier, physical or sensory disability, and limited health literacy but reported similar receipt of publicly funded home and community care in the post-discharge period (Table 1).

**Table 1:** Comparison of Baseline and Discharge Demographics Between Income Groups (*N*=443)

Variable	*N*=443 *n* (%)	Low income *n*=111 *n* (%)	Not low income *n*=115 *n* (%)	Non-responders to income category *n*=217 *n* (%)
Female sex	235 (53.0)	59 (53.2)	56 (48.7)	120 (55.3)
Mean age ± SD	70.1 ± 15.5	70.1 ± 13.5	75.4 ± 14.4	68.6 ±16.6
Age ≥ 65 years	307 (69.3)	88 (79.3)	78 (67.8)	141 (65.0)
Admission diagnosis:				
CHF	125 (28.2)	43 (38.7)	31 (27.0)	51 (23.5)
Pneumonia	97 (21.9)	31 (27.9)	30 (26.1)	36 (16.6)
COPD	69 (15.6)	25 (22.5)	24 (20.9)	20 (9.2)
Orthopedic surgery	152 (34.3)	12 (10.8)	30 (26.1)	110 (50.7)
Language barrier	76 (17.2)	35 (31.5)	7 (6.1)	34 (15.7)
Limited health literacy	159 (36.0)	66 (59.5)	26 (22.8)	67 (30.9)
Physical disability	184 (41.5)	70 (63.1)	45 (39.1)	69 (31.8)
Sensory disability	128 (28.9)	46 (41.4)	37 (32.2)	45 (20.7)
Living alone	135 (30.6)	44 (39.6)	37 (32.2)	54 (25.1)
Education high school or less	108 (24.4)	54 (48.7)	16 (13.9)	38 (17.9)
Not born in Canada	193 (43.7)	58 (52.7)	40 (34.8)	95 (43.8)
Help from family:				
With self-care	125 (28.2)	40 (36.0)	28 (24.3)	57 (26.3)
With food preparation	163 (36.8)	39 (35.1)	41 (35.7)	83 (28.2)
With medications	93 (21.0)	27 (24.3)	21 (18.3)	45 (20.7)
With transportation	164 (37.0)	43 (38.7)	37 (32.2)	84 (38.7)
With attending appointments	157 (35.4)	44 (39.6)	38 (33.0)	75 (34.6)
No help from family	205 (46.3)	48 (43.2)	55 (47.8)	102 (47.0)
Discharge support (e.g., home care) requested	212 (48.1)	66 (60.0)	59 (51.8)	130 (59.9)
Discharged with diet restrictions	86 (19.5)	28 (25.5)	20 (17.4)	38 (17.5)
Discharged with activity restrictions	141 (31.9)	21 (19.1)	33 (28.7)	87 (40.1)
Discharged with follow-up appointments	432 (98.0)	107 (97.3)	111 (97.4)	214 (98.6)
Patient-reported receipt of home care	172 (38.8)	42 (37.8)	47 (40.9)	83 (38.2)

Self-reported income was not found to be associated with patient experience responses. Patient adherence to discharge plans such as primary care follow-up, medication, and diet and activity restrictions was also similar across income groups. While individuals who reported low income had lower proportions of follow-up with specialists when compared to those with higher income (57.6% vs. 70.9%), these were not statistically significant. Lastly, there were very similar unscheduled ED visits, readmission, or death between the two income groups when measured at 1 and 3 months post-discharge (Table 2). Results from our adjusted analyses showed similar results (Table 2). In our quantitative analyses, there was no significant effect modification of language barrier, physical disability, and social isolation on income for any of our post-discharge outcomes.

**Table 2:** Unadjusted and Adjusted Odds Ratio of Post-discharge Outcomes Between Low and Higher Income Groups^*^

Variable	Low income *n*=111 *N* (%)	Not low income *n*=115 *N* (%)	Unadjusted OR (95% CI)	Adjusted OR (95% CI)
Patient experience during care transition:				
Discussion about help needed	73 (65.8)	68 (59.1)	1.3 (0.7–2.4)	1.0 (0.6–1.9)^§^
Received information in writing	58 (52.3)	59 (51.3)	1.0 (0.6–1.8)	1.2 (0.7–2.2)^‡^
Clear understanding of medication	75 (68.2)	65 (56.5)	1.6 (0.9–3.0)	1.9 (1.0–3.4)^‡^
Information about what to do if worried	52 (47.3)	48 (41.7)	1.3 (0.7–2.2)	1.4 (0.8–2.7)^‡^
Better understanding of condition	53 (48.2)	54 (47.0)	1.0 (0.6–1.8)	1.3 (0.7–2.4)^‡^
Clear understanding of follow-ups	70 (63.6)	68 (59.1)	1.2 (0.7–2.1)	1.2 (0.6–2.2)^‡^
Adherence to instructions at 1 month post-discharge:				
Adherence to medications^†^	94 (94.0)	99 (92.5)	1.3 (0.4–4.6)	-
Adherence to diet restrictions^†^	25 (92.6)	19 (95.0)	0.7 (0.01–13.6)	-
Adherence to activity restrictions^†^	24 (88.9)	42 (93.3)	0.6 (0.07–4.6)	-
Adherence to follow-up with family doctor	76 (76.0)	72 (75.0)	0.9 (0.5–1.9)	0.9 (0.4–1.9)^‖^
Adherence to follow-up with specialist	38 (57.6)	56 (70.9)	0.6 (0.3–1.2)	0.6 (0.3–1.5)^‖^
Unscheduled healthcare utilization:				
Unexpected ER visits, readmission or death at 1 month post-discharge	27 (24.5)	29 (25.7)	0.9 (0.5–1.8)	0.9 (0.5–1.9)^‖^
Unexpected ER visits, readmission or death at 3 months post-discharge	29 (27.4)	30 (27.3)	1.0 (0.5–1.9)	1.0 (0.5–2.0)^‖^

^*^Reference group = higher income group

^†^Adherence outcomes are unadjusted due to too few events making adjusted measures unstable. Random site effect added to models

^‡^Adjusted for age, sex, admission diagnosis, limited health literacy, language barrier, lack of family involvement, ^§^and physical disability

^‖^Adjusted for age, sex, admission diagnosis, limited health literacy, lack of family involvement, physical disability, and discharge support

Approximately half (*n*=217) of our participants did not know or preferred not to report their income status. These individuals had lower proportions of self-reported sociodemographic disparities such as language barriers or physical disability. When we added this category to adjusted models, a noticeable difference was found (Appendix D). Low-income individuals and those who preferred not to answer on income were however more likely to report understanding their medications completely when compared to higher income individuals (OR 1.9, 95% CI: 1.1–3.4, *p*=0.037 and OR 1.7, 95% CI: 1.1–2.9, *p*=0.029).

### Qualitative Results

Twenty-five of 63 participants (40%) interviewed following discharge from hospital had cost or income-relevant quotes which were included in the qualitative analysis. While demographics among those interviewed were similar to the overall cohort used for quantitative analysis, only 24% of those interviewed self-reported low income and 52% chose not to respond to income category.

**Figure 2:**
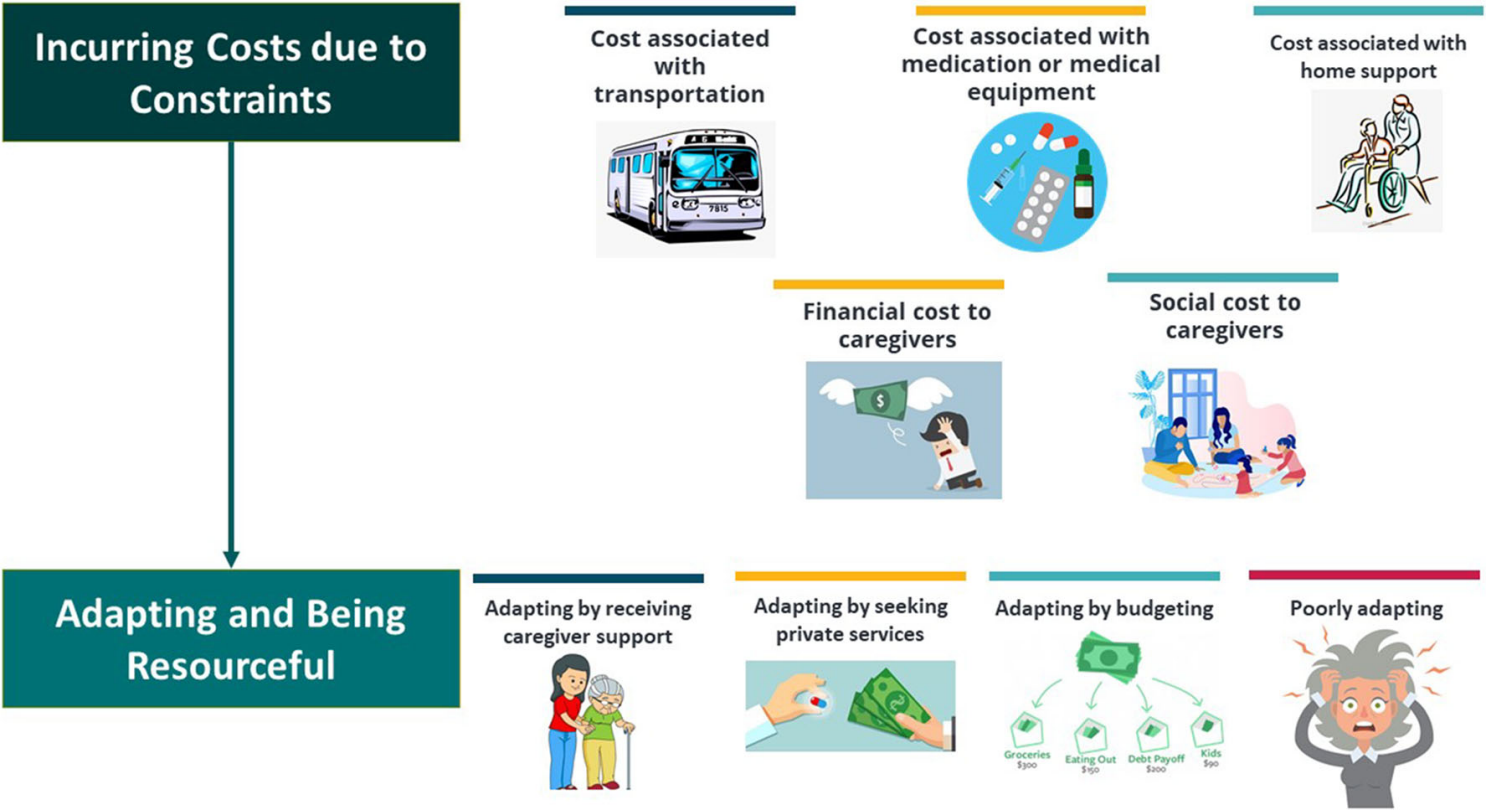
Illustration of identified qualitative themes.

We identified two intersecting themes: (1) incurring costs due to constraints of publicly funded services and (2) adapting and being resourceful, including a subset of patients and caregivers who are unable to adapt well (Fig. 2). Additional narrative excerpts aligned with these two themes are described below (Table 3).

**Table 3:** Exemplary Quotes for Each Major Theme Identified from the Qualitative Strand

Themes	Representative quote (participant ID, patient/caregiver)
Incurring costs due to constraints	“I am trying to arrange a lift to get back to [name of hospital where surgery occurred] on the 17th of November for my first check up through them, but I found out it’s a bit expensive. It’s not free but at least it’s available if I can’t get someone else to take me because I’m not allowed to drive of course.” (15, patient, income unknown)
	“I can’t walk from my bathroom to my bedroom to my kitchen. Now, it is a large condominium, but you know, I can’t go into my kitchen and start making meals. So, you know, even with an hour and a half of [publicly covered community home care] [would be helpful]. I’m waiting on [an] organization for dependent services which I don’t pay for because I’m paying a fortune right now. And I was fortunate, I had a paid…PSW... and she brought me home...in a cab...and sort of...and made sure I ate. And she tidied up.” (10, patient, higher income)
	“So, effectively, the message is basically that [name of hospital] wants to get you out as fast as they can because of the costs, and then you’re on the hook for the costs in a retirement home until such time as you can basically remodel your house or until the person is physically able to actually cope in the house.” (18, patient, income unknown)
Adapting and being resourceful	“I’ve got community support. I’ve got [a] homemaker. I’ve got PSW’s” (02, patient, low income)
	“I have to pay for it so I just... try to budget other things.” (06, patient, higher income)
	“I wasn’t paying much attention to that because of my job today, that was driving a truck, long distance, and after I lost my first job, there wasn’t much time to take care of this, and I never checked on my heart apart from the mandatory medical checks from the ministry of transportation which happens every three years.” (05, patient, income unknown)
	“Had he gone home, he wasn’t well enough to run around getting tests. I would have had to take another week off work, and I may never have gotten him into respite care.” (17, caregiver, patient low income)
	“My husband probably would have been able to do it, but he had so much to do that he didn’t.” (19, patient, income unknown)

### Incurring Costs due to Constraints of Publicly Funded Services

During the transition process, respondents highlighted various additional costs not covered by publicly funded services for transportation, medications or medical equipment, and home care supports. Patients spoke of inadequate, unreliable, or lack of clarity about public home care to meet their needs, which then led to additional costs for themselves and/or their family caregivers. Referred to as CCAC, the province’s public community care access centers at the time, one patient responded: “I’ve even got a church member who will go and buy my groceries. Because it didn’t seem that CCAC was available for that. It turns out, for the longest time they had that service and they’ve now cut it. And to go to appointments, the same thing. I did not know that I could ask for an escort. And now CCAC, I’ve discovered now, that they have cut it.” (02, low income patient). Individuals with physical disability voiced additional costs needed to meet home care, equipment, or transportation needs. Some individuals spoke of challenges in getting to appointments or picking up medications, which led to additional costs for transportation or additional help in the home not covered through public services. One respondent voiced, “I couldn’t sweep my floors. I couldn’t vacuum. How do you make a meal with one hand?” (07, low income patient). And another expressed, “I’ve got personal support workers put in place so that at the end of their time with me, I’m in the wheelchair with a coat and I’m going out the door with them. So that’s five days a week. If I want to do anything on a Saturday or a Sunday, they don’t have as many people…If they are going to come, they will come when you see them…I don’t do a lot of things on weekends.” (02, low income patient).

Patients and caregivers also expressed financial costs for the caregiver. “You know, my wife’s home too as she’s trying to take care of me and I don’t know if that’s a good thing because we need money to survive, pay bills and mortgages” (01, patient, income unknown). Another caregiver respondent mentioned how their ability to assist their family member is limited by how much time they are able to take off from work. “Having said that, I was averaging a day a week because of all his medical appointments. And that was interfering with my ability to earn a living.” (17, caregiver of low income patient). Additionally, when caregivers supported their family members by conducting IADLs including “going to the grocery store … cleaning his house … doing his laundry,” they witnessed losing the opportunity to engage in the relationship presenting as a social cost to caregivers. A caregiver shared the social cost of conducting IADLs for their loved one as limiting the time to “go and visit my father and have quality time with him.” (17, caregiver of patient with low income).

### Varying Levels of Adapting and Being Resourceful

Several patients spoke of the transition back to normalcy following discharge, voicing concerns over employment, leading to some patients adapting by prioritizing finances over their health. A patient noted, “On one hand, I feel like I shouldn’t be rushing that much to get back to work, but on the other hand I have clients that need followed up on and if I don’t follow up on them, then someone else will and I lose out on the commission” (08, patient, income unknown). The fear of losing employment or income made the respondent claim that “I was still calling some of them from the hospital.” (08, patient, income unknown).

Patients also adapted through supplementing public system support with caregiver support, private services, and through other various means such as budgeting or saving equipment for future use. For instance, one respondent voiced, “Thank God I have a sister who helps me. Because like when I first came home, I couldn’t do anything” (07, low-income patient). Whereas, other patients sought private services to meet their needs, as one patient claimed, “No, its private [physiotherapy], at [a different location]. I wouldn’t go back to [name of hospital he was an inpatient], let’s put it this way.” (16, higher income patient). Similarly, some patients found it helpful to budget to stretch their income to ensure all necessities are paid for, to hold on to equipment that was expensive in case it was needed later, or to choose cheaper food options in order to keep costs down. “I’ve been sorting out money, making sure I have enough money in the bank to pay the rent” (04, higher income patient). Not all patients were able to adapt, with some just accepting it, and as a result, described the transition process as, “I am having a hard time [coping] with everything if I am honest with you” (05, patient, income unknown). Support from family, friends, or community members was more commonly reported among individuals with low income or those who preferred not to respond to income category than individuals in the higher income group. Individuals in the lower income group often mentioned not worrying about having money to pay for medications due to government subsidies.

## DISCUSSION

We conducted a mixed-methods secondary analysis among individuals enrolled in a randomized controlled trial focused on improving the quality of patient instructions on patient experience. We evaluated the association between low income and post-discharge health outcomes and explored patient and caregiver perspectives on the role of income disparities and costs following discharge from hospital. Our study found few quantitative differences between the post-discharge health outcomes of individuals with low income when compared to individuals with higher income. Our qualitative results however highlight numerous costs that exist for patients and their families following a care transition to home which may not have been identified quantitatively based on income category alone. First, transportation costs, out-of-pocket medication, medical equipment, and unreliable home care services were cited barriers to recovery. Furthermore, caregivers incurred social costs and lost wages due to their caregiving responsibilities. Our study findings also highlighted three main adaptation strategies which was reported with slightly higher frequency among our low income and unknown income categories (budgeting, receiving caregiver support, and seeking private services) which may help explain how patients and caregivers offset constraints in a publicly funded system.

This study has notable limitations. First, this study took place among a selective group of patients with medical and orthopedic conditions receiving healthcare and public services across one province in Canada (Ontario) with the majority of participants residing in the city of Toronto. As health care and services are delivered at a provincial level, identified challenges and limitations may vary in other areas of Canada. Second, individuals were enrolled from an RCT which explored the impact of a written discharge summary with patient-oriented instructions on patient experience. While the intervention was equally provided to individuals from all income classes and did not find differences to patient experience nor adherence to follow-up appointments and further healthcare use, it is certainly possible healthcare-seeking differences may have not been captured and led to selection bias in our study. Third, the qualitative component of the mixed-methods study is designed to explore rather than to test a hypothesis, and further studies are warranted to clarify if those with caregivers or additional private support may differ from individuals without these supports. Lastly, 24% of those who were interviewed were in the high income household category, which may have underestimated the true costs and impact of these costs on individuals with low SES. Our focus on costs beyond solely SES and wide range of words captured in our interviews is a strength of our study. While themes identified are similar to other studies, further research with a larger sample among individuals with self-reported low income or other measures of financial instability in a public-payer system such as Canada’s is needed.^5^ Strengths of our study include the mixed-methods design which allowed a richer evaluation of hidden costs during the post-discharge period and the use of self-reported income, rather than neighborhood income, which can provide a more accurate picture of a patient’s financial status.^26^

There are several potential explanations why we did not find an association between income and the studied patient outcomes. It is possible that low-income individuals in our study had different health-seeking behaviors which were not captured in our study. Recent efforts in Ontario have worked to ensure new care interventions and models of care support patient and caregiver engagement and higher quality communication at time of discharge.^4^ Several quality improvements have also centered on improving medication reconciliation and understanding, which might explain why individuals with low-income had a better understanding of their medications.^30^ Nevertheless, our study elucidates the many out-of-pocket additional costs (e.g., medications, transportation, and home support) which are not always covered by publicly funded coverage and which fall under the responsibility of the individual patient or family caregiver once discharged home. These findings are in line with another recently published study that highlighted unreliable, untimely, or inadequate home care and out-of-pocket medication costs which affected adherence to medications changes after hospital discharge.^26^ First, it is possible that the ascertainment of low income was insufficient in our study. We had a high proportion of non-response to our income question (almost 50%) which is higher than reported in a growing number of studies.^11, 26^ The use of neighborhood income may lead to misclassification bias and bias towards the null; this is because there can be differences in the interpretation of “the number of people supported” and estimation to self-reported income questions.^31^ Our study echoes these concerns and calls for further work to better measure financial strain during the post-discharge period. Recent Canadian studies have found that upstream effects such as housing, food insecurity, isolation, or lack of social supports including income assistance or provincial disability pensions may be better markers of income instability and help explain how socioeconomic constraints impact post-discharge behaviors.^11^ Second, our cohort was generally an older but well-supported group of individuals. More than half of our cohort had family caregivers that supported them with post-discharge activities, only 40% who lived alone, and almost unanimously all had follow-up plans with primary care providers. Prior studies have found living alone and poor satisfaction with primary care to be important risk factors for early hospital readmission in low-income older adults.^32^

Our findings add to the current literature by sharing important insights on how patients and caregivers may adapt by prioritizing finances and relying on their support networks when transitioning from hospital to home. These findings are important, as little attention up to now has been given to the costs to patients and their families during care transitions in a public-payer system such as Canada. The financial and social burden that caregivers experience is an important area for further research and intervention particularly given what is known around the essential role of caregivers in the post-discharge period.^33^ The most effective discharge interventions include caregivers longitudinally as active care partners throughout the discharge process, especially during the education, medication counseling, and planning of outpatient follow-up.^5, 34–36^

## CONCLUSION

Our study identified few differences in patient experience, adherence to instructions, or unscheduled visits and death up to 3 months following discharge from hospital between individuals who reported low income and those that reported higher income. There are nevertheless substantial costs for patients and their families not always covered by public funding. A better understanding of these financial challenges is needed so that patients can receive the necessary support to manage their post-discharge recovery regardless of costs.

## Supplementary Information

ESM 1 (DOCX 28 kb)
