# Supplementary material for: “I Had Bills to Pay”: a Mixed-Methods Study on the Role of Income on Care Transitions in a Public-Payer Healthcare System
Source: J Gen Intern Med. 2023 Jan 25;38(7):1606–14. doi: 10.1007/s11606-023-08024-7 (PMC10212904; doi:10.1007/s11606-023-08024-7)
Supplement: Supplementary file 1 — (DOCX 28 kb) [file 11606_2023_8024_MOESM1_ESM.docx]

# Appendix A. Distribution of Study Population Using Statistics Canada Definition of Low Income (N=111)

|  | **Number of individuals the income supports** | | | | | | |  |  |
| --- | --- | --- | --- | --- | --- | --- | --- | --- | --- |
| **Total family income** | **1** | **2** | **3** | **4** | **5** | **≥ 6** | **Missing** | **Total number of individuals who reported income** | **Number of individuals in low income group** |
| $0 to $29,000 | 91 | 15 | 0 | 2 | 0 | 0 | 0 | 108 | 108 |
| $30,000 to $59,000 | 36 | 20 | 1 | 2 | 0 | 0 | 0 | 59 | 3 |
| $60,000 to $89,000 | 9 | 13 | 0 | 0 | 0 | 0 | 0 | 22 | 0 |
| $90,000 to $119,999 | 5 | 4 | 1 | 1 | 1 | 1 | 0 | 13 | 0 |
| $120,000 to $149,000 | 4 | 3 | 1 | 0 | 0 | 0 | 0 | 8 | 0 |
| $150,000 or more | 7 | 9 | 0 | 0 | 0 | 0 | 0 | 16 | 0 |
| Total | - | - | - | - | - | - | - | 226 | 111 |

*Shaded rows are considered individuals in low income group^23^

# Appendix B. CPES-IC Survey Questions Used

| **Topic** | **Question** | **Response Options** |
| --- | --- | --- |
| Discharge planning | 1) During this hospital stay, did doctors, nurses or other hospital staff talk with you about whether you would have the help you needed when you left the hospital? | Yes, No |
|  | 2) During this hospital stay, did you get information in writing about what symptoms or health problems to look out for after you left the hospital? | Yes, No |
| Discharge management | 3) Before you left the hospital, did you have a clear understanding about all of your prescribed medications, including those you were taking before your hospital stay? | Not at all, Partly, Quite a bit, Completely, Not applicable |
|  | 4) Did you receive enough information from hospital staff about what to do if you were worried about your condition or treatment after you left the hospital? | Not at all, Partly, Quite a bit, Completely |
|  | 5) When you left the hospital, did you have a better understanding of your condition than when you entered? | Not at all, Partly, Quite a bit, Completely |
|  | 6) When you left the hospital, did you have a clear understanding about your follow-up appointments and investigations? | Disagree, Strongly disagree, Agree, Strongly agree |

# Appendix C. Demographics between individuals included in study cohort (N=443) versus lost to follow up or no-longer interested individuals at 72 hours post discharge (N=50)

| **Variable** | **Study cohort**  **n (% of Total)**  **N = 443** | **Individuals who left at 72 hours**  **n (% of Total)**  **N = 50** |
| --- | --- | --- |
| Female sex | 235 (53.1) | 23 (46.0) |
| Age ≥ 65 years | 307 (69.3) | 30 (60.0) |
| Admission diagnosis CHF | 125 (28.2) | 20 (40.0) |
| Admission diagnosis pneumonia | 97 (21.9) | 14 (28.0) |
| Admission diagnosis COPD | 69 (15.6) | 9 (18.0) |
| Admitted for orthopedic procedure | 152 (34.3) | 5 (10.0) |
| Language barrier | 73 (16.5) | 12 (24.0) |
| Limited health literacy | 159 (36.0) | 13 (26.0) |
| Physical disability | 184 (41.5) | 15 (30.0) |
| Sensory disability | 128 (28.9) | 11 (22.0) |
| Living alone | 135 (30.6) | 10 (29.4) |
| Education less than high school | 108 (24.4) | 9 (18.0) |
| Below low-income cut-off | 111 (49.1) | 13 (26.0) |
| Immigrant or refugee | 193 (43.7) | 19 (38.0) |

# Appendix D. Adjusted Odds Ratio of Post-Discharge Outcomes Between low income and non-responder to income category Groups^*^

| **Variable** | **Low income**  **n=111**  **N (%)** | **Non-responders to income category**  **n=217**  **N (%)** | **Adjusted OR (95% CI)** |
| --- | --- | --- | --- |
| Patient experience during care transition: |  |  |  |
| Discussion about help needed | 73 (65.8) | 151 (65.9) | 0.7 (0.4-1.32)^§^ |
| Received information in writing | 58 (52.3) | 133 (61.3) | 0.9 (0.6-1.6)^‡^ |
| Clear understanding of medication | 75 (68.2) | 152 (70.0) | 1.1 (0.6-1.9)^‡^ |
| Information about what to do if worried | 52 (47.3) | 96 (44.2) | 1.2 (0.7-2.1)^‡^ |
| Better understanding of condition | 53 (48.2) | 95 (43.8) | 1.5 (0.9-2.4)^‡^ |
| Clear understanding of follow-ups | 70 (63.6) | 125 (57.6) | 1.2 (0.7-2.0)^‡^ |
| Adherence to instructions at 1 month post-discharge: |  |  |  |
| Adherence to medications^†^ | 94 (94.0) | 183 (94.3) | - |
| Adherence to diet restrictions^†^ | 25 (92.6) | 32 (91.4) | - |
| Adherence to activity restrictions^†^ | 24 (88.9) | 98 (97.0) | - |
| Adherence to follow-up with family doctor | 76 (76.0) | 157 (82.2) | 0.6 (0.3-1.2)^‖^ |
| Adherence to follow-up with specialist | 38 (57.6) | 86 (66.2) | 0.7 (0.4-1.5)^‖^ |
| Unscheduled health care utilization: |  |  |  |
| Unexpected ER visits, readmission or death at 1 month post-discharge | 27 (24.5) | 48 (22.6) | 1.0 (0.5-1.7)^‖^ |
| Unexpected ER visits, readmission or death at 3 months post-discharge | 29 (27.4) | 44 (21.2) | 0.7 (0.4-1.5)^‖^ |

^*^Reference group = non-responders group

^†^Adherence outcomes are unadjusted due to too few events making adjusted measures unstable. Random site effect added to models.

^‡^Adjusted for age, sex, admission diagnosis, limited health literacy, language barrier, lack of family involvement ^§^and physical disability

^‖^Adjusted for age, sex, admission diagnosis, limited health literacy, lack of family involvement, physical disability and discharge support
